# Specific Bacterial Taxa and Their Metabolite, DHPS, May Be Linked to Gut Dyshomeostasis in Patients with Alzheimer’s Disease, Parkinson’s Disease, and Amyotrophic Lateral Sclerosis

**DOI:** 10.3390/nu17091597

**Published:** 2025-05-06

**Authors:** Courtney Jayde Christopher, Katherine Hope Morgan, Christopher Mahone Tolleson, Randall Trudell, Roberto Fernandez-Romero, Lexis Rice, Blessing A. Abiodun, Zane Vickery, Katarina A. Jones, Brittni Morgan Woodall, Christopher Nagy, Piotr Andrzej Mieczkowski, Gregory Bowen, Shawn R. Campagna, Joseph Christopher Ellis

**Affiliations:** 1 Department of Chemistry, University of Tennessee, Knoxville, TN 37996, USA; cleathe3@vols.utk.edu (C.J.C.); babiodun@vols.utk.edu (B.A.A.); zvickery@vols.utk.edu (Z.V.); brittniwoodall@gmail.com (B.M.W.); campagna@utk.edu (S.R.C.); 2 College of Nursing, University of Tennessee, Knoxville, TN 37996, USA; kmorgan3@utk.edu; 3 The Cole Center for Parkinson’s and Movement Disorders, The University of Tennessee Medical Center, Knoxville, TN 37922, USArtrudell@utmck.edu (R.T.); 4 The Pat Summitt Clinic, The University of Tennessee Medical Center, Knoxville, TN 37920, USA; 5 Department of Biochemistry & Cellular and Molecular Biology, University of Tennessee, Knoxville, TN 37996, USA; lrice13@vols.utk.edu; 6 Biological and Small Molecule Mass Spectrometry Core, University of Tennessee, Knoxville, TN 37996, USA; kjone166@vols.utk.edu; 7 High Throughput Sequencing Facility, School of Medicine, University of North Carolina at Chapel Hill, Chapel Hill, NC 27514, USA; cnagy@email.unc.edu; 8 Department of Genetics, School of Medicine, University of North Carolina at Chapel Hill, Chapel Hill, NC 27514, USA; piotr_mieczkowski@med.unc.edu; 9 Integrated Genomics Cores, University of North Carolina at Chapel Hill, Chapel Hill, NC 27514, USA; greg_bowen@med.unc.edu; 10 NetEllis, LLC, Knoxville, TN 37934, USA; 11 Department of Medicine, School of Medicine, University of Tennessee Graduate, Knoxville, TN 37996, USA; 12 Biosciences Division, Oak Ridge National Laboratory, Oak Ridge, TN 37830, USA

**Keywords:** neurodegenerative disease, amyotrophic lateral sclerosis, Parkinson’s disease, Alzheimer’s disease, metabolomics, bacterial-derived, 2,3-dihydroxypropane-1-sulfonate (DHPS), microbiome

## Abstract

**Background:** Neurodegenerative diseases (NDDs) are multifactorial disorders frequently associated with gut dysbiosis, oxidative stress, and inflammation; however, the pathophysiological mechanisms remain poorly understood. **Methods:** Using untargeted mass spectrometry-based metabolomics and 16S sequencing of human stool, we investigated bacterial and metabolic dyshomeostasis in the gut microbiome associated with early disease stages across three NDDs—amyotrophic lateral sclerosis (ALS), Alzheimer’s disease (AD), Parkinson’s disease (PD)—and healthy controls (HC). **Results**: We discovered a previously unrecognized link between a microbial-derived metabolite with an unknown role in human physiology, 2,3-dihydroxypropane-1-sulfonate (DHPS), and gut dysbiosis in NDDs. DHPS was downregulated in AD, ALS, and PD, while bacteria involved in DHPS metabolism, *Eubacterium* and *Desulfovibrio*, were increased in all disease cohorts. Additionally, select taxa within the Clostridia class had strong negative correlations to DHPS, suggesting a potential role in DHPS metabolism. A catabolic product of DHPS is hydrogen sulfide, and when in excess, it is known to promote inflammation, oxidative stress, mitochondrial damage, and gut dysbiosis, known hallmarks of NDDs. **Conclusions**: These findings suggest that cryptic sulfur metabolism via DHPS is a potential missing link in our current understanding of gut dysbiosis associated with NDD onset and progression. As this was a hypothesis generating study, more work is needed to elucidate the role of DHPS in gut dysbiosis and neurodegenerative diseases.

## 1. Introduction

The social and economic impact of neurodegenerative diseases (NDDs), such as Alzheimer’s (AD), amyotrophic lateral sclerosis (ALS), and Parkinson’s (PD), has devasting impacts on patients and their families alike. Worldwide, NDDs are the leading cause of disability [1], reduced lifespan and quality of life, and are the second leading cause of death [2]. ALS is usually fatal within four to five years of diagnosis. The economic burden in the United States (U.S.) is over USD 51 billion annually for PD, [3]; over USD 1 billion annually for ALS [4]; and USD 360 billion for AD and related dementias in 2024 [5]. Despite decades of research, the cause of NDD, broadly, and of ALS, AD, PD, specifically, remains poorly understood.

In humans and in animal models, AD, ALS, and PD are associated with irregularities in the gut microbiome and its metabolic byproducts (the metabolome), compared to healthy controls (HCs) [6,7,8,9,10,11,12,13,14,15,16,17,18,19]. Imbalances in the gut microbiome and metabolome can be greatly influenced by extrinsic epigenetic factors (e.g., chemical exposure) [20]. The human gut hosts millions of gut microbes whose community composition and metabolic byproducts influence human health and may play a significant role in mediating or promulgating NDDs [21,22,23,24], including inflammatory responses in the body and the brain [25]. Others have recently compared the gut (fecal) metabolomics across these three diseases [26]. Of particular interest in the present study was the identification of metabolites produced solely by gut microbiota, and not by the human host, called microbial-derived metabolites. We sought to identify microbial-derived metabolites shared by AD, ALS, and PD, with the potential to identify a common pathway that influences neurodegeneration. Microbial-derived metabolites may yield clues about how the microbiome influences NDDs, contributes to biomarker discovery for earlier diagnosis, and helps identify potential interventions in abnormal metabolic processes that will slow disease progression.

### 1.1. Microbial and Metabolic Dysbiosis Associated with NDDs

#### 1.1.1. Alzheimer’s Disease

AD is a progressive neurodegenerative disorder characterized by the deposition of extracellular aggregates of amyloid-beta 42 (Aβ-42) and intraneuronal hyperphosphorylated tau (p-tau) [27,28]. The accumulation of amyloid and tau are believed to be early events in a cascade of pathological processes that may be modulated by genetic factors [29] and mediated by such mechanisms as neuroinflammation [30] and synaptic dysfunction. Over time, this chain of events will inevitably result in neurodegeneration, progressive cognitive decline, and untimely dementia [31]. Significant differences in AD gut metabolome have been noted in AD mice compared to HC [32,33,34]. Also, in mice, fecal microbiota transfer of healthy microbiota has been found to decrease AD pathologies [35]. In their review article, Liang, Pereira [36] recently summarized how the human AD gut microbiome differs from HC, particularly affecting abundances of bacterial phyla Actinobacteria, Bacteroidetes, Firmicutes, Proteobacteria, and Varrucomicrobia in several different studies [37,38,39,40,41,42,43]. Shifts in microbial diversity in AD have demonstrated increases in proinflammatory genera and decreases in anti-inflammatory bacteria [36]. The gut metabolome of AD also differs from HC for short-chain fatty acids (SCFAs), tryptophan metabolites, and lithocholic [15], as well as for organic acids, lipids and lipid-like molecules, organic nitrogen, and pathways affecting steroid hormone synthesis, N-acyl amino acid metabolism, and piperidine metabolism [44]. Differences in volatile organic compounds (VOCs) occur between AD and HC stool metabolomics, which vary with the degree of AD advancement [14].

#### 1.1.2. Amyotrophic Lateral Sclerosis

ALS is a progressive motor neuron disorder characterized by death of a variable combination of “upper” and “lower” motor neurons, causing increasing weakness and spasticity of voluntary limb and bulbar function. The gut microbiome of ALS has more Bacteroidetes and less Firmicutes at the phylum level, also less of genus *Megamona*, and these changes are accompanied by altered metabolic pathways in ALS compared to HC [17]. Due to increased abundances in several studies of people with ALS, Bacteroidetes and *Escherichia* are suspected to play an influential role in ALS [26]. Comparatively fewer studies have been published on the fecal metabolome of ALS. In one metabolomic study of people with ALS with cognitive impairment, a significantly altered bile acid profile was observed compared to people with ALS who had normal cognition [16]. In another study, altered microbial abundances were significantly associated with metabolomic changes such as increased propanoic acid, 3,7-dihydroxy-12-oxochoanoic acid, and coproporphyrinogen I, and 4-hydroxybenzoylcholine, while a decrease was found in acylcarnitine [17]. In the first case study of a female ALS patient who underwent washed microbial transplants, researchers measured improvements in ALS symptomology over 11 months, concurrent with changes in the microbiome and metabolomics [45].

#### 1.1.3. Parkinson’s Disease

PD is diagnosed clinically by several well-defined criteria (e.g., bradykinesia, resting tremor, and rigidity) [46]. While it is known that pathologically PD is characterized by the degeneration of dopamine neurons in the substantia nigra pars compacta, as well as Lewy body intracellular inclusions largely composed of the protein α-synuclein, the underlying cause or causes for PD are not known [47,48]. It appears to be secondary to a variety of interrelated factors with the gut and inflammatory mechanisms continuing to garner increasing interest [47,49]. It is well documented that the gut microbiome and metabolome of PD differ significantly from controls [50]. For example, in PD patients on dopaminergic treatment, bacterial taxa associated with anti-inflammatory or neuroprotective metabolites were reduced in an Italian cohort study and linked to changes in the metabolomic profile, namely in lipids, vitamins, and amino acids [10]. *Actinobacteria*, *Proteobacteria*, and *Verrucomicrobia* were significantly increased compared to HC, while significant decreases were noted in *Bacteroidetes* and *Cyanobacteria* [10]. The PD microbiome seems to be characterized by a shift towards pathogenic members of Proteobacteria phylum, such as Enterobacteriaceae, with the decreased expression of the members of the phyla Firmicutes and Bacteroides: these shifts are associated with increased inflammation. Dysbiosis in the PD fecal metabolome has been the subject of several studies [6,8,9,10,11,12,19,51], including correlations between clinical phenotypes of PD and gut inflammation [6].

### 1.2. Gut Imbalances, Inflammation, Oxidative Stress, and Mitochondrial Dysfunction

The gut is in constant system-wide communication with organs throughout the body [52], and dyshomeostasis within the metabolome can contribute to intestinal and systemic inflammation, oxidative stress, and mitochondrial dysfunction, which are involved in the pathology of NDDs [53]. For example, hydrogen sulfide (H_2_S) is a key metabolite involved in sulfur metabolism with a myriad of beneficial physiological functions, as reviewed by Hansen and Venkatachalam [54]. In the human gut microbiome, H_2_S is primarily produced by members of the *Desulfovibrio* and *Bilophila* species [55,56,57]. However, endogenous excess H_2_S promotes inflammation [58,59,60], oxidative stress [61], mitochondrial damage [62], and gut dysbiosis [56,63]. Dyshomeostasis in sulfur metabolism has been well documented in PD [7,64,65], and Munteanu et al. [66] have highlighted the known and potential roles of excess H_2_S production in AD pathology. The host–microbe regulation of oxidized sulfur compounds in the metabolome is gaining recognition in health and disease [54,67].

The objective of this study was to identify bacterial and metabolic signatures in the human gut associated with three NDDs and HC by conducting 16s rRNA sequencing and untargeted metabolomics in a cross-sectional cohort analysis. To explore microbial and metabolomic imbalances relatively early in the progression of these three NDDs, we recruited patients early in their disease from their respective treating neurology subspecialists. Our purpose was to discover potentially shared alterations in microbial community structure and metabolomic signatures in these three NDDs that may serve as biomarkers for earlier diagnosis and potential interventions.

## 2. Materials and Methods

### 2.1. Ethics Statement

This study was approved by the institutional review board (IRB) of the University of Tennessee Health Science Center for the protection of human subjects (IRB#4535).

### 2.2. Study Population

Participants were recruited during their first or second visit to a movement disorder specialist for ALS, AD, or PD who were living in the 21-county regional service area of the University of Tennessee Medical Center, in Knoxville, Tennessee. Participation was voluntary, and only those who were willing and able to complete the study activities (with a caregiver if needed) were included. Participants were excluded if they had received antibiotic or systemic antifungal medications in the previous six months or had a history of bowel cancer or bowel disease. Further inclusion criteria for each cohort were as follows: (1) For AD: 65 years of age or older, met all clinical criteria for mild-to-moderate AD according to the National Institute on Aging-Alzheimer’s Association criteria [68], were otherwise in good health based on medical history and clinical examination; (2) ALS: age of 30 years or older at enrollment, and met the El Escorial Criteria [69] for definite, probable, or possible ALS; (3) PD: aged 50 to 85 years at enrollment, diagnosed within the last 5 years, absence of PD medications with the exception of MAO-B inhibitors, absence of dyskinesia or motor fluctuations, modified Hoehn and Yahr Stage [70] <2.5 (symptoms ranging from unilateral involvement only [Stage 1] to mild bilateral involvement with recovery on a pull test [Stage 2.5]); and (4) HC: aged 30 or older, who were age matched (±4 years) and sex-matched, and in good health based on medical history and clinical examination. HC aged 65 years or older completed the Neuropsychological Battery from the National Alzheimer’s Coordinating Center [71] prior to enrollment to demonstrate normal cognition.

This study took place in South Central Appalachia in East Tennessee of the United States. On 33 out of 41 health indicators, performance in the rural Appalachian Region is worse than the national performance, including seven of 10 leading causes of death (heart disease, cancer, chronic obstructive pulmonary disease, injury, stroke, diabetes, and suicide) [72]. Mortality rates are higher than the national rates in Appalachia, yet there have been few studies conducted in Appalachia. This study offers data and provides insight from an underrepresented and unique region of the USA.

### 2.3. Subjects Were in Early Diagnostic Stages

Participants were recruited early in disease progression (See Table 1 for additional demographics) at neurology clinics of the University of Tennessee Medical Center in Knoxville, Tennessee. Participants were non-Hispanic Caucasians (100%). None of the participants had taken antibiotic or systemic antifungal medications in the previous six months. None had a history of bowel cancer or bowel disease. Healthy controls > 65 years of age demonstrated normal cognition without signs of dementia on cognitive tests. The ALS and PD cohorts were not tested for normal cognition. From summer 2021 to spring 2022, 48 participants enrolled, and 45 completed the study. Of these, 40 submitted stool samples (AD = 5, ALS = 11, PD = 13, and HC = 11).

### 2.4. Sample Collection, Preservation, DNA Extraction

Samples were collected at home by participants within one week of enrollment. Participants were instructed to place stool from the same specimen into two vials as follows: (1) the DNA Genotek^®^ gut microbiome DNA collection kit (DNA Genotek, Ottawa, ON, Canada) and (2) a 50-mL sterile tube, which was immediately frozen at home at −20 °C. Stool samples were delivered frozen on ice by same-day courier service, to the University of Tennessee College of Nursing’s biobehavioral laboratory for nucleic acid extraction. Samples were stored at −80 °C.

From the DNA Genotek^®^ kits, DNA extraction was conducted in batches, using Zymo’s *Quick*-DNA Fecal/soil Microbe Kit (Zymo Research, Irvine, CA, USA). Extractions were frozen at −80 °C, then shipped on dry ice to the University of North Carolina (UNC) Chapel Hill High Throughput Sequencing Facility (HTSF) for 16s rRNA/DNA sequencing.

From the 50-mL sterile tubes, approximately 50 mg of stool was aliquoted to sterile tubes, kept frozen at −80 °C, then transferred to the Biological and Small Molecule Mass spectrometry Core at the University of Tennessee Knoxville (BSMMSC) for metabolomics extractions and analysis.

### 2.5. Metabolomics

#### 2.5.1. Metabolite Extractions

All samples were extracted and analyzed at the BSMMSC at the University of Tennessee Knoxville (RRID: SCR_021368). Stool samples were kept at 4 °C, pre-weighed, and homogenized. The exact mass of stool used for sample was recorded and used for normalization. Roughly 50 mg of stool was aliquoted, and water-soluble metabolites were extracted using an acidic acetonitrile extraction procedure adapted from Rabinowitz and Kimball [73], using 1.5 mL of 4:4:2 acetonitrile–methanol–water with 0.1 M formic acid [74,75]. All solvents were HPLC grade. The supernatant collected from each sample was dried under nitrogen, then 300 µL of LC-MS grade water was added to each sample before mass spectral analysis.

#### 2.5.2. UHPLC-HRMS

A previously described ultra high-performance liquid chromatography high resolution mass spectrometry (UHPLC-HRMS) method was used for untargeted metabolomics analysis [76], using an UltiMate 3000 RS autosampler (Dionex, Sunnyvale, CA, USA), Synergi 2.6 µm Hydro RP column (100 mm × 2.1 mm, 100 Å; Phenomenex, Torrance, CA, USA), an UltiMate 3000 pump (Dionex), and Exactive Plus Orbitrap mass spectrometer (Thermo Fisher Scientific, Waltham, MA, USA). A previously described 25 min gradient elution, reverse phase ion-paring method with a water–methanol solvent system, and a tributylamine ion pairing reagent was used for chromatographic separation [77]. Metabolites were ionized via negative mode electrospray ionization (ESI) prior to full scan mass spectral analysis as previously described [76].

#### 2.5.3. Metabolomics Data Processing

Raw mass spectral files were converted to mzML files using a package from ProteoWizard, msConverter (v 3.0.2) [78]. All mzML files were imported into an open-source software, metabolomics analysis, and visualization engine (El-MAVEN, v0.12.0), where metabolites were manually identified using an in-house library based on exact mass (±5 ppm) and retention time (±2 min) [74,79,80]. Metabolite peaks were integrated and raw peak intensities for identified metabolites were exported from El-MAVEN to a csv file. Prior to statistical analysis, raw spectral data were normalized by mass of sample used for extraction.

#### 2.5.4. Statistical Analysis

The normalized data were imported into MetaboAnalyst 5.0 and were filtered via interquartile range (IQR), log transformed, and Pareto scaled [81,82,83]. Partial least squares discriminant analysis (PLS-DA) was performed in MetaboAnalyst 5.0, where variable importance in projection (VIP) scores were assigned to each metabolite to indicate the importance of each metabolite in contributing to the separation between experimental groups. VIP scores > 1 indicate that a metabolite significantly contributes to the separation of groups in the PLS-DA model. Venn diagrams were constructed using metabolites with VIP scores >1 from pairwise group comparisons. MetaboAnalyst 5.0 was also used to generate volcano plots to visualize metabolites significantly different between groups.

### 2.6. 16s rRNA Sequencing

In this study, we implemented a detailed two-step protocol for 16S rRNA gene amplification [84] prioritizing high precision and reproducibility across numerous samples. The initial PCR reaction was meticulously configured, utilizing specific volumes of Kapa Enhancer, Kapa Buffer A (KAPA Robust 2G-Roche), forward and reverse Frame Shift Molecular Tags (FSMT) primers (Table 2), and other necessary components, culminating in a total reaction volume of 50 µL. The use of KAPA Robust 2G in the initial PCR reaction is necessary to prevent artifacts caused by non-specific extension from imperfectly annealed primers. The FSMT primers, comprising six frames for both 338F and 806R types, were carefully mixed. To streamline the handling of multiple samples, a ‘ready-mix’ comprising essential Kapa components was prepared. This strategy was instrumental in reducing batch effects, particularly when utilizing reagents from different Kapa kits. The ready-mix was then allocated into aliquots, each adequate for a 96-well plate. The first PCR cycle’s conditions were meticulously programmed to include specific durations and steps for denaturation, annealing, and extension, set at 95 °C for one minute, followed by 10 cycles of 95 °C for 15 s, 50 °C for 30 s, and 72 °C for 30 s, then 72 °C for one minute to complete extension for all molecules, and a final indefinite hold at 4 °C. Post-PCR, bead purification was performed to selectively exclude DNA fragments shorter than 300 bp, employing a 0.68:1 bead-to-DNA ratio. The second nested-PCR phase (indexing) followed a similar methodology but with modifications in the reaction components, such as the Kapa HiFi Readymix (KAPA HiFi-Roche, Basel, Switzerland) and specific Adaptor_primer1 and indexing primers. The cycling conditions for this phase were set at 95 °C for one minute, followed by 22 cycles of 95 °C for 15 s, 60 °C for 30 s, and 72 °C for 30 s, concluding with 75 °C for one minute and an indefinite hold at 4 °C. Finally, for 16S sequencing, we used a series of custom-designed primers, chosen for their specificity and amplification efficiency. The sequences of these primers, along with the custom sequencing primer (NextF_Read1_seq), are comprehensively detailed in the Supplementary Materials. Prepared libraries were pooled and sequenced on MiSeq Pair End 2 × 300 v2 flowcell (Table 2). We generated a minimum of 120,000 paired reads per sample for analysis.

#### 16S Data Processing and Analysis

16S amplicon sequencing was performed using the moving pictures documentation in QIIME2 [85]. Briefly, sequences were imported into QIIME2 (version 2023.2). Paired-end sequences were denoised with DADA2. Trimming values were set; p-trim-left was set to 20, p-trim-len was set to 220, and sampling depth was set at 120,000 sequences (Figures S8–S10). Finally, an OTU table was produced for downstream analysis. Traditional microbial ecology analyses, such as alpha diversity and beta diversity, were also performed. In addition, taxonomic classification of the reads was performed in the QIIME2 using Silva 138 99% OTUs full-length sequences.

Relative abundances from operational taxonomic units (OTUs) were analyzed on the phylum, class, order, family, and genus levels. QIIME taxonomy labels with corresponding abundances were uploaded to MicrobiomeAnalyst, and the data were scaled by total sum prior to statistical and meta-analysis of microbiome data [83]. Alpha diversity was accessed at the genus level by Chao, Shannon, and Simpson indexes, with the Mann–Whitney test used to determine if alpha diversity differed across cohorts. Stacked bar graphs were used to visualize OTU relative abundances across cohorts. Pairwise comparisons between cohorts were made using the statistical method EdgeR. OTU correlations were analyzed using either Pearson’s r or Spearman’s rho correlation coefficients.

## 3. Results

### 3.1. Global Metabolomics

**Figure 1:**
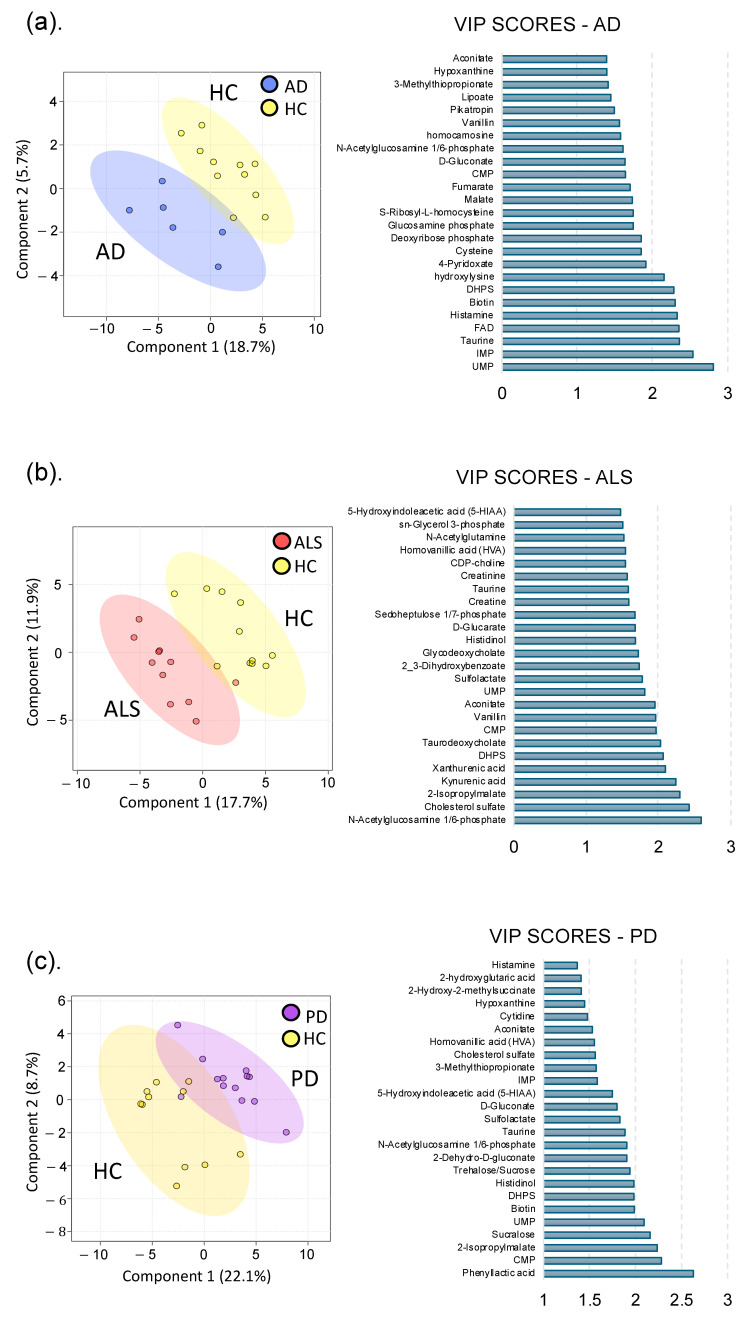
Patients with NDDs have an altered gut metabolic profile. Partial least squares discriminant analysis (PLS-DA) comparing metabolomes associated with NDD and HC and corresponding variable importance in projection (VIP) scores. Individual participants are shown with respective confidence intervals; HC samples are yellow; (**a**) AD samples are blue; (**b**) ALS samples are shown in red; (**c**) PD samples are purple. VIP scores are shown for the corresponding PLS-DA models showing the top 25 metabolites contributing most to the differences in metabolic profiles.

Untargeted metabolomics was used to investigate functional alterations in the gut metabolome associated with NDDs. We identified 164 metabolites based on exact mass and retention time and evaluated metabolic profile differences between each NDD and HC using PLS-DA, which revealed that each NDD had a unique gut metabolic profile compared to HC (Figure 1). To determine metabolites altered by NDD, metabolites from each pairwise comparison with a fold change >|1.5| (*p* < 0.1) or variable importance in projection (VIP) score >1 were used for further analysis (See Table S4 for a comprehensive list and Supplementary Data for fold change, *p* values, and VIP scores). These metabolites were also used to identify unique metabolic signatures for each NDD. This revealed 14, 20, and 9 unique markers for AD, ALS, and PD, respectively. Moreover, we identified 19 metabolic markers, which were consistently altered across all three NDDs in this study.

#### 3.1.1. Alzheimer’s Disease

**Figure 2:**
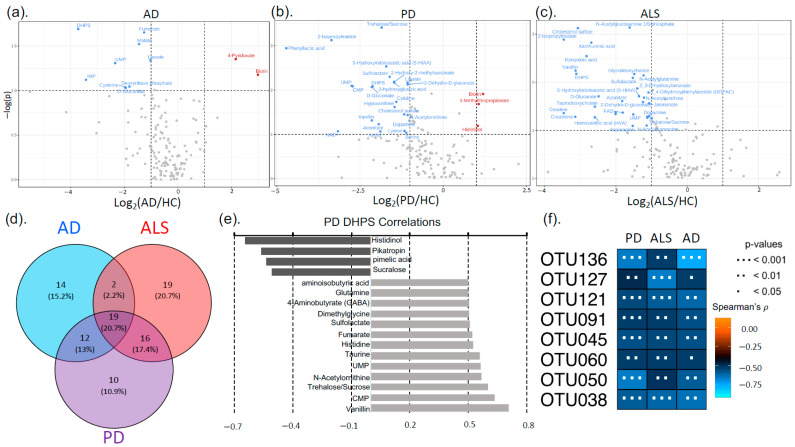
There were 19 metabolic markers for all NDDs in this study, as well as unique markers for each disease. DHPS was one of the 19 markers for NDDs, as it was significantly decreased in NDD cohorts. DHPS was strongly correlated to acetylated amino acids and taurine. (**a**–**c**) Volcano plots showing metabolites significantly different between cohorts. The *x*-axis is the log_2_ foldchange and *y*-axis is the -log *p* value. The following cutoffs were used: fold change > |1.5| (*p* < 0.1). (**d**) Venn diagram shows unique and shared metabolic markers. These metabolites had fold change > |1.5| (*p* < 0.1) or VIP score > 1. (**e**) Metabolites strongly correlated with DHPS in PD. (**f**) Eight OTUs were significantly correlated with DHPS across all three NDDs. These OTUs were the following: OTU136 (d__Bacteria;p__Firmicutes;c__Clostridia;__;__;__); OUT127 (d__Bacteria;p__Firmicutes;c__Clostridia;o__Clostridia;f__Hungateiclostridiaceae;__); OTU121 (d__Bacteria;p__Firmicutes;c__Clostridia;o__Oscillospirales;f__Oscillospiraceae;g__*Oscillospira*); OTU091 (d__Bacteria;p__Firmicutes;c__Clostridia;o__Oscillospirales;f__UCG-010;g__*UCG-010*); OTU045 (d__Bacteria;p__Firmicutes;c__Clostridia;o__Clostridia_vadinBB60_group;f__Clostridia_vadinBB60_group;g__*Clostridia_vadinBB60_group*); OTU060 (d__Bacteria;p__Bacteroidota;c__Bacteroidia;o__Bacteroidales; f__Marinifilaceae;g__*Odoribacter*); OTU050 (d__Bacteria;p__Firmicutes;c__Clostridia;o__Oscillospirales;f__Oscillospiraceae;__); and OTU038 (d__Bacteria;p__Firmicutes;c__Clostridia;o__Oscillospirales;f__[Eubacterium]_coprostanoligenes_group;g__*[Eubacterium]_coprostanoligenes_group*).

Based on the PLS-DA model between the AD and HC metabolomes, the overall metabolic profile of the gut in AD patients was distinctly different from HC, as seen by the clustering and separation of groups (Figure 1). Metabolites with the highest VIP scores, contributing most to global metabolome differences, were uridine monophosphate (UMP) (2.8), inosine monophosphate (IMP) (2.5), and taurine (2.4). We observed that 29% of identified metabolites in the gut were altered by AD, and 14 of these metabolites were unique to AD. Notably, the primary catabolic product of vitamin B_6_ (4-pyridoxate), S-ribosyl-L-homocysteine, 3-methylphenylacetic acid, and metabolites involved in fatty acid and energy metabolism (carnitine, hydroxylysine, FMN) were unique to AD. Metabolic profile differences in AD demonstrated altered sulfur metabolism, with downregulation of cysteine, taurine, methionine, homocysteine, and DHPS, while sulfur containing B vitamins, biotin and pyridoxine, were increased in the AD cohort compared to HC (Figure 2 and Figure S2 for altered AD stool metabolic pathways).

#### 3.1.2. Amyotrophic Lateral Sclerosis

Similarly, the ALS cohort also exhibited global gut metabolome differences compared to HC, as shown in Figure 1. Metabolites contributing most to differences between cohorts were N-acetylglucosamine phosphate (VIP = 2.6), cholesterol sulfate (VIP = 2.4), 2-isopropylmalate (VIP = 2.3), and kynurenic acid (VIP = 2.2) (Figure 2). Overall, 34% of identified metabolites were significantly altered in the ALS cohort: of these, histadinol and CDP-choline were increased, while all other metabolites were decreased. Similar to AD, we observed that ALS caused alterations in tryptophan metabolism, sulfur metabolism, and acetylated amino acids in the gut (Figure 2, see also Figure S3 for altered ALS stool metabolic pathways). There were 19 metabolites unique to ALS, including metabolites involved in tryptophan metabolism (xanthurenic acid, kynurenic acid), vitamin B_6_ (pyridoxine), and energy, fatty acid, and amino acid metabolism (creatine, creatinine, arginine, CDP-choline, and glycodeoxycholate). Interestingly, creatine metabolism was unique to the ALS cohort, with no notable alterations in AD or PD cohorts.

#### 3.1.3. Parkinson’s Disease

The most dramatic alterations in the gut metabolome were found when comparing the PD to HC cohort, with 35% of identified metabolites being altered by PD (Figure 1). Phenyllactic acid (2.6), CMP (2.3), 2-isopropylmalate (2.3), sucralose (2.2), and UMP (2.1) had the highest VIP scores, contributing most to the global metabolome differences between the PD and HC cohorts in the PLS-DA model. Similar to both AD and ALS, PD caused alterations in sulfur, tryptophan, vitamin B, and acetylated amino acid metabolism (see Figure S4 for pathways altered in the PD stool metabolome). Other notable differences in the PD gut metabolome include downregulation of taurine, alanine, nicotinate and nicotinamide, and cysteine and methionine metabolism (Figure 2). Conversely, biotin, sucralose, 3-methylthiopropionate, and histidinol were upregulated in PD (Figure 2). There were 10 unique metabolic markers associated with PD, including xylose, lysine, xylitol, and N-acetyl-beta-alanine. The most dramatic alterations in neurotransmitters (5-Hydroxyindoleacetic acid (5-HIAA), dopamine, 3,4-dihydroxyphenylacetate (DOPAC)) were observed in the gut metabolome of PD patients.

### 3.2. Neurodegenerative Disease Metabolic Markers

There were 19 metabolites altered by the three NDDs in the study, serving as metabolic markers for AD, ALS, and PD (Figure 2 and Table S5). Many of these metabolites were involved in sulfur and vitamin B metabolism (DHPS, taurine, 3-methylthiopropionate, cholesterol sulfate, sulfolactate, and biotin). DHPS is a microbial-derived metabolite and was on average 9× lower (*p* < 0.05) in the NDD cohorts compared to the HC cohort (Figure 2). We also observed that DHPS had strong correlations to acetylated amino acids and metabolites involved in neurotransmitter, bile acid, and vitamin B metabolism (Figure 2; also see Figure S1 and Supplementary Data S4 for metabolites correlated with DHPS in NDDs).

### 3.3. Sequencing (16s rRNA)

**Figure 3:**
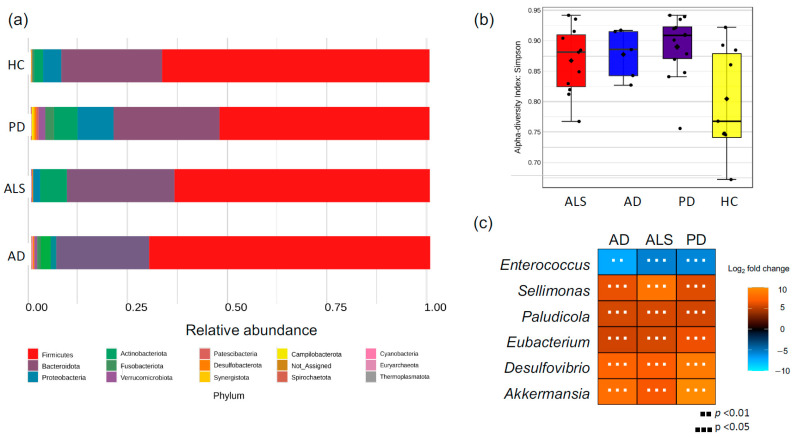
Gut microbial composition is altered in NDDs. (**a**) Phyla-level differences in microbial composition. (**b**) Box and whisker plot showing alpha diversity was significantly higher in PD cohort compared to the HC cohort. However, there were no significant differences in the AD and ALS cohort compared to HC. (**c**) Log_2_ fold change of taxa significantly altered across AD, ALS, and PD cohorts. Orange represents higher abundance in disease compared to HC, while blue represents lower abundance in disease compared to HC.

The microbial composition of stool samples from NDD and HC patients was characterized via 16S amplicon sequencing. Differences in alpha diversity (Figure 3 and Figure S5) were only observed when comparing the PD and HC cohorts, with PD having higher alpha diversity (*p* = 0.02). The analysis of OTUs revealed phylum-level differences in the abundance of Firmicutes in PD (53%) compared to HC (67%) stool, while Firmicutes displayed minimal differences in ALS and AD cohorts (Figure 3). Phylum Actinobacteria was unchanged in AD but increased in PD and ALS compared to HC patients. Differences within the Firmicutes phylum in PD could broadly be attributed to genus *Eubacterium*. There were six taxa significantly altered across all NDDs compared to HCs: *Desulfovibrio, Eubacterium*, *Akkermansia*, *Paludicola*, *Sellimonas*, and *Enterococcus* (Figure 3, Figures S6 and S7, and Supplementary Data). All of these taxa were more abundant in the NDD cohorts than HC, except for *Enterococcus*, which was higher in HC.

To assess the relationship between gut microbial composition and function in NDDs, we identified OTUs correlated with metabolites that were significantly different in NDDs. Microbial-derived metabolite DHPS was consistently and significantly altered in NDDs and there were 33, 43, and 15 OTUs significantly correlated with DHPS in PD, AD, and ALS, respectively, all having negative correlations (see Supplementary Data S4). Of these, eight OTUs were correlated with DHPS across PD, AD, and ALS, and all were part of the Firmicutes phylum (class *Clostridia*), except for one belonging to the Bacteroidetes phylum (Figure 2).

## 4. Discussion

### 4.1. Metabolome Alterations in NDDs

While it has become widely accepted that gut dysbiosis is a contributing factor in NDDs, the pathophysiological mechanism remains elusive. This is, in part, due to the complexity of the gut microbiome and metabolome and “dark matter” in host–microbiome interactions. This “dark matter” represents the vast amount of detected, but unidentified, small molecules in the metabolome. These analytes likely impact human physiology, highlighting the need for better understanding the small molecules involved in host–microbiome interactions in the gut. To this end, we used an untargeted, hypothesis-generating approach to investigate gut microbiome alterations in NDDs.

As expected, we observed differences in the gut microbiome composition and function in patients with NDDs (AD, ALS, and PD) compared to HCs. The presented data align with previous findings in the literature that sulfur metabolism, tryptophan catabolism, bile acids, and amino acids are altered in NDDs [9,86,87,88]. We identified 19 consistent markers for AD, ALS, and PD, as well as metabolic signatures unique to each NDD.

Carnitine was a metabolic signature unique to AD and is known to play a crucial role in energy metabolism and mitochondrial function. Dietary-carnitine supplements have been proposed for prevention or alleviation of AD [89]. In ALS, creatine and creatinine were unique signatures and decreased in ALS. Creatine has been proposed to exhibit protective effects against mitochondrial dysfunction and may also be neuroprotective as it has been linked to increased survival time in an ALS clinical trial [90].

Two of the unique metabolic signatures for PD were decreased xylose and xylitol, indicating impaired sugar metabolism. Xylose and xylitol feed into the pentose phosphate pathway (PPP) resulting in the production of NADPH, pyruvate, and shikimate, which are building blocks for some branched chain and aromatic amino acids and antioxidants. This result is compelling since neurons preferentially use the PPP for sugar metabolism and rely on the metabolic products of the PPP for protection against oxidative stress [91]. Decreased flux through the PPP results in reduced antioxidant capacity and increased oxidative stress, which is a leading hypothesis for pathogenesis of PD [92].

The 19 metabolic markers for NDD showed significant alterations in sulfur, cholesterol, sugar, and vitamin B metabolism. To our surprise, one of these markers was DHPS, a sulfonated microbial-derived metabolite known for its role in sulfur and carbon flux in marine ecosystems, with an unknown role in human physiology [93,94]. With this, we have discovered a novel link between microbial-derived metabolite DHPS and NDDs, as DHPS was a significant driver of global metabolome differences when comparing NDD and HC cohorts. To the best of our knowledge, we are the first to provide data linking DHPS to three NDDs—AD, ALS, and PD—thereby demonstrating a potential role of DHPS in human physiology.

### 4.2. Cryptic Sulfur Metabolism in NDDs

All detected metabolites in the transsulfuration pathway were decreased in NDD cohorts, except for cystathionine. Rapid conversion of homocysteine to cystathionine could suggest increased fluxes in sulfur metabolism, as previous work using longitudinal metabolome data and constraint-based modeling of gut microbial communities has shown increased flux in the transsulfuration pathway in PD [7]. This pathway generates glutathione and H_2_S, which impact redox metabolism. Increased fluxes through sulfur metabolism may be indicative of excess H_2_S which, at low concentrations, is neuroprotective and cytoprotective. However, at high levels, H_2_S induces mitochondrial damage [62], oxidative stress [61], systemic inflammation [58,59,60], and gut dysbiosis [56,63]. Although we do not have direct results on mitochondrial bioenergetics, our findings may support the current hypothesis that the increased burden of oxidative stress caused by mitochondrial dysfunction leads to neuroinflammation [95]. It has been documented that NDDs share the common feature of defective endoplasmic reticulum (ER)–mitochondria signaling that disrupts the proper function of mitochondria and contributes to pathology of NDDs. Disruptions to effective signaling occur where regions of the ER closely associate with mitochondria through mitochondria-associated membranes (MAMS) as well as proteins [96]. Mitochondria play critical roles in cellular function, not only as the powerhouses of the cell, but also in maintaining Ca^2+^ homeostasis, apoptosis, lipid synthesis, cell growth and differentiation, and the production of reactive oxidative species [53,97,98,99].

In addition to metabolites in the transsulfuration pathway, other sulfur-containing metabolites, DHPS and taurine, were significantly altered across NDDs. We found this to be intriguing as oxidized sulfur-containing small molecules represent an underappreciated class of unique, biologically relevant metabolites in host–microbiome interactions [67]. It has been discovered recently that these molecules play a significant role in cellular signaling and impact disease-related phenotypes, thereby bringing oxidized sulfur metabolites to the forefront of enhancing our understanding of the pathophysiological impact of host–microbiome interactions [67,100,101,102,103]. Since the role of DHPS in human physiology and NDDs is unknown, we identified metabolites and OTUs significantly correlated with DHPS. Five metabolites (taurine, vanillin, histidinol, *N*-acetylornithine, and sucrose/trehalose), and eight OTUs were significantly (*p* < 0.01) correlated with DHPS in each NDD cohort. All of the significantly correlated OTUs belonged to phylum Firmicutes (orders Clostridia and Oscillospirales) except for one, which belonged to the phylum Bacteroidetes (genus *Odoribacter*). All of these microbes have been directly linked to metabolizing dietary sulfur to H_2_S [104,105]. Together, the metabolites and OTUs correlated with DHPS suggest that DHPS is involved in cryptic sulfur host–microbiome metabolism in NDDs.

### 4.3. DHPS Microbial Metabolism in NDDs

Frommeyer and colleagues annotated genes required for a novel bacterial transaldolase pathway by which select gut microbes (*Enterococcus*, *Clostridium*, *Desulfovibrio*, and *Eubacterium* strains) can ferment dietary sulfoquinovose (SQ) to H_2_S with DHPS as a transient intermediate [56]. SQ is obtained through the intake of leafy greens and vegetables containing the lipid sulfoquinovosyl diacylglycerol (SQDG), which is a highly abundant sulfur-containing metabolite common in photosynthetic plants and cyanobacteria. In addition, Hanson and colleagues [56] reported that DHPS contributes to intestinal H_2_S production, and that *Eubacterium rectale* (*E. rectale*) is the primary DHPS producer, while *Desulfovibrio* and *Bilophila* are the primary DHPS consumers, in fecal microcosms from human vegetarians. They identified genes necessary for microbial catabolism of diet-acquired SQ to H_2_S and analyzed the expression of these genes across stool metatranscriptomes of 123 healthy, 28 ulcerative colitis, and 50 Crohn’s disease patients to identify the overall impact on health [56]. However, they found no significant differences in the expression of these pathways between cohorts with bowel disease and healthy individuals [56].

*Eubacterium rectale* plays a significant role in promoting inflammation and damaging tissue in the gut [106]. We found that the genus *Eubacterium* was increased in PD, ALS, and AD, and it was one of the eight OTUs strongly correlated with DHPS across all three diseases (Figure 3). Additionally, sulfate-reducing bacteria (SRB), such as *Desulfovibrio*, generate H_2_S, and have previously been implicated in the development and severity of PD [64]. Here, we observed *Desulfovibrio* were significantly altered in PD, ALS, and AD and were more abundant in patients with NDDs (Figure 3). Additionally, taurine was strongly correlated with DHPS and significantly decreased in NDDs and can also be obtained through dietary sources and degraded by *Desulfovibrio and Bilophilia* to acetyl-CoA and H_2_S. Other studies have also implicated *Bilophilia wadsworthia* in PD altering microbial sulfur metabolism, yet the etiology remains elusive [7,9,107,108]. Disrupted sulfur metabolism in NDDs could result in increased flux through DHPS and taurine, promoting overgrowth of SRB and excess H_2_S. This may explain why DHPS-producing and -consuming microbes are increased in NDDs, while DHPS is decreased.

While we do not have dietary information from participants, it has become fairly accepted that the gut microbiome composition and function in NDDs are influenced by dietary habits and nutrition [109]. Since diet-acquired SQDG from leafy greens can be bioconverted to DHPS by select microbes in the gut, we hypothesize that diet corresponds to DHPS and sulfur homeostasis in NDDs. Given this, it is imperative to investigate the outcomes of a plant-based diet in NDDs patients.

### 4.4. Melainabacteria Could Be Associated with DHPS-Mediated Cryptic Sulfur Metabolism in NDDs

In 2013, a novel class of microbes, Melainabacteria, were discovered in the human gut [110]. Melainabacteria are a non-photosynthetic clade of cyanobacteria and their role in the human gut and physiology is largely unknown [110,111,112,113]. This is of particular interest in our study as SQDG is highly abundant in cyanobacteria. The abundance of Melainabacteria in gut is correlated with diet as the highest abundances of Melainabacteria in stool have been found in herbivore populations, with lower abundances in omnivore and carnivore populations suggesting that Melainabacteria have a role in fermenting dietary plant fibers [110]. This may also provide another connection of DHPS to diet.

Furthermore, a partial metabolic reconstruction from Soo et al. (2014) of *Gastranaerophilales*, one of the six major taxonomic orders within the Melainabacteria class, is predicted to use the Embden–Meyerhof pathway to acquire energy by fermenting simple carbohydrates, and DHPS was positively correlated with trehalose in this study suggesting a link between DHPS and *Gastranaerophilales* [112]. This metabolic reconstruction also revealed that *Gastranaerophilales* have the genes required for the biosynthesis of B vitamins (biotin, riboflavin, nicotinamide, and dihydrofolate) and vitamin K, which could demonstrate a beneficial relationship between Melainabacteria and humans [110,112,114]. The basis for the symbiotic relationship between *Thalassiosira pseudonana* and Roseobacters in aquatic systems was vitamin B_12_ and DHPS [93]. Given that Melainabacteria are suggested to have a syntrophic H_2_-producing niche, it is likely that these microbes can degrade dietary sulfonates. In addition, a previous study has shown that *Gastranaerophilales* is one of the main producers of microbial-derived indole leading to increased indole in PD [115] We found that DHPS was negatively correlated with both biotin and metabolites involved in indole metabolism. The majority of preliminary studies show a trend of increased Melainabacteria abundance in patients with metabolic, neurodegenerative, and gastrointestinal disease, though results in the literature are still inconsistent, as understanding the role of *Gastranaerophilales* in the human gut remains in its infancy [114,116]. In this study, we found that the abundance of *Gastranaerophilales* was increased in PD, ALS, and AD compared to the HC cohort. This contradictory evidence highlights the need to further investigate the role of Melainabacteria. In addition, *Gastranaerophilales* was strongly correlated to *Desulfovibrio.* This may suggest that *Gastranaerophilales* plays a role in DHPS metabolism since *Desulfovibrio* has the cellular machinery to both produce and degrade DHPS. With this, we propose there could be a relationship between Melainabacteria and DHPS, given the phylogenetic similarity to cyanobacteria and the negative correlation between DHPS and biotin.

### 4.5. Potential Physiological Role of DHPS

Previous studies have laid the foundation for investigating DHPS in human metabolism by elucidating the biochemical mechanism of DHPS production and degradation in the human gut and directly linking dietary sulfonates to H_2_S production [56,94,105]; however, the data did not demonstrate an association between DHPS, or the genes involved in DHPS metabolism, and human health outcomes. Recently, Zaparte, Christopher and colleagues [117] provided the first report of direct DHPS detection in human stool samples and highlighted a compelling connection between DHPS and gut metabolic dysregulation. This study investigated the systemic impact of e-cigarette and/or combustible tobacco/marijuana smoking in people living with HIV (PLWH). They observed that DHPS in human stool samples was significantly higher (20×) in the PLWH control cohort compared to the smoking cohorts and noted strong correlations between DHPS and vitamin B, acetylated amino acids, bile acids, and cholesterol metabolism. Both e-cigarette use/smoking and NDDs are associated with gut dysbiosis and inflammation [118]. Although strictly correlative in both studies, DHPS was significantly increased in the control cohorts and decreased in the cohorts associated with gut dysbiosis. This conserved trend across independent datasets may indicate that DHPS abundance in human stool is associated with overall health status or gut dysbiosis and inflammation, rather than being unique to NDDs. Therefore, we hypothesize that DHPS is a missing link in the pathophysiology of inflammation, as it may be a key regulator of gut inflammation and overall human health.

### 4.6. Limitations

This study lays the foundation for future discoveries on the role of specific bacterial taxa and their metabolite DHPS; however, there are some notable limitations. First, all patients were sampled in the same geographic location of Eastern Tennessee and the surrounding Appalachian Mountains, and recruitment during the pandemic was slow, resulting in small sample sizes, particularly in the AD population, who were not seen in person for several months during the pandemic, which explains the lower age distribution of the AD cohort. In addition, all the patients were Caucasian. Combined, this limits the generalizability of the conclusions. Future studies should include measures of dietary intake to better investigate the role of diet and its influence on DHPS production, as well measuring plasma markers of inflammation to see how peripheral inflammation may relate to DHPS. Additionally, DHPS not being included in the metabolite library used for analyte identification is likely why other NDD and human health studies have not reported similar implications of DHPS. Given this, we encourage others to mine previously analyzed datasets for DHPS and include DHPS in the metabolite reference library for future studies to aid in elucidating the physiological role of DHPS. While this study describes a remarkably well-conserved and novel association across different neurodegenerative diseases, this study is limited by not being able to define these findings as causative, given the exploratory nature of untargeted metabolomics.

## 5. Conclusions

**Figure 4:**
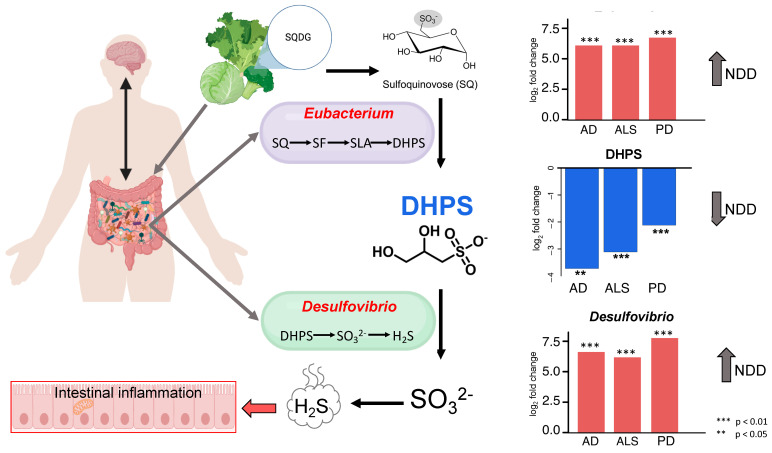
DHPS can be obtained through dietary consumption of leafy greens and degraded to H_2_S. The abundance of DHPS-producing and -consuming microbes is increased in NDD, while DHPS is decreased in NDD, suggesting the rapid degradation of DHPS to yield H_2_S. Other microbes (*Enterococcus* and *Clostridium*) can ferment dietary sulfoquinovose (SQ) to H_2_S with DHPS as a transient intermediate as well. Figure was created using biorender.com and adapted from Hanson et al. [56].

In this study, we investigated bacterial and metabolic dyshomeostasis in the gut associated with AD, ALS, and PD using an integrative cross-omics approach. Herein, we present the first linkage of DHPS, a microbial-derived metabolite, to gut dysbiosis associated with NDDs, which may contribute to dyshomeostasis in sulfur metabolism. DHPS-consuming and -degrading microbes are more abundant in NDDs, while DHPS is decreased (Figure 4). We hypothesize that the decrease in DHPS in NDDs may be a result of increased flux through the recently identified transaldolase pathway in which dietary sulfonates are metabolized to DHPS and H_2_S by select gut microbes, including *Eubacterium* and *Desulfovibrio*. Excess H_2_S, resulting from increased flux through DHPS, may contribute to mitochondrial dysfunction, oxidative stress, inflammation, and gut dysbiosis in NDDs. While there is substantial literature on the physiological impacts of H_2_S [108,119,120], we have reported a novel link among DHPS, gut dyshomeostasis, and NDDs. These findings highlight the need for further investigation into the role of cryptic sulfur metabolism via DHPS, as DHPS may serve as a missing link in our current understanding of gut dysbiosis and its association with NDD onset and progression. In conclusion, this study lays the groundwork for developing DHPS-based diagnostic tools and microbiome-modulating therapies to detect and potentially intervene in the early stages of neurodegenerative diseases.

## Figures and Tables

**Table 1:** Demographics of study participants.

	ALS	AD	PD	HC
Number	11	5	13	11
Age in years mean (range)	72.5 (71–74)	64.5 (43–77)	65.5 (51–80)	66.1 (45–87)
Female	4	3	9	8
Male	8	2	4	6
BMI mean (range)	25.4 (17.7–28.8)	27.7 (19.2–37.8)	28.3 (22.4–39.5)	30.1 (20–58.4)
Taking medications for NDD	69%	100%	7%	0
Months from symptom onset: average (range)	27.2 (6–82)	35.7 (30–117)	31.6 (15–57)	
Months from diagnosis: average (range)	16.9 (1–64)	18.3 (9–68)	3 (0–20)	
MoCA score (average)		19.8		26.5
Clinical Dementia Rating Scale raw (average)		2.75		0
Clinical Dementia Rating Scale global (average)		0.42		0
ALS bulbar	23%			
ALS spinal	77%			
UPDRS-part I (average)			6.9	
UPDRS-part II (average)			6.7	
UPDRS-part III (average)			33.9	
UPDRS-part IV (average)			0	
UPDRS gastrointestinal symptoms (average)			0.23	
Hoehn and Yahr			2.0	

ALS = amyotrophic lateral sclerosis, AD = Alzheimer’s disease, and PD = Parkinson’s disease; MoCA = Montreal Cognitive Assessment, where 18–25 = mild cognitive impairment, 10–17 = moderate, and <10 = severe cognitive impairment; NDD = neurodegenerative disease; UPDRS = Unified Parkinson’s Disease Rating Scale. The Clinical Dementia Rating Scale Global tracks a patient’s level of impairment/dementia, where 0 = normal, 0.5 = very mild dementia, 1 = mild dementia, 2 = moderate dementia, and 3 = severe dementia.

**Table 2:** 16s Primers and sequences.

Primer	Sequence
338F_f1_bc1	TCCCTCGCGCCATCAGAGATGTGTATAAGAGACAGNNNNTGANNNNTCACTCCTACGGGAGGCAGCA
338F_f2_bc1	CCCTCGCGCCATCAGAGATGTGTATAAGAGACAGNNNNTTGANNNNTCACTCCTACGGGAGGCAGCA
338F_f3_bc1	TCCCTCGCGCCATCAGAGATGTGTATAAGAGACAGNNNNCTTGANNNNTCACTCCTACGGGAGGCAGCA
338F_f4_bc1	TCCCTCGCGCCATCAGAGATGTGTATAAGAGACAGNNNNACTTGANNNNTCACTCCTACGGGAGGCAGCA
338F_f5_bc1	TCCCTCGCGCCATCAGAGATGTGTATAAGAGACAGNNNNGACTTGANNNNTCACTCCTACGGGAGGCAGCA
338F_f6_bc1	TCCCTCGCGCCATCAGAGATGTGTATAAGAGACAGNNNNTGACTTGANNNNTCACTCCTACGGGAGGCAGCA
JMPM_806R_C	GTGACTGGAGTTCAGACGTGTGCTCTTCCGATCTNNNNCTAGGACTACHVGGGTWTCTAAT
JMPM_806R_2T	GTGACTGGAGTTCAGACGTGTGCTCTTCCGATCTNNNNTCTAGGACTACHVGGGTWTCTAAT
JMPM_806R_2A	GTGACTGGAGTTCAGACGTGTGCTCTTCCGATCTNNNNATCTAGGACTACHVGGGTWTCTAAT
JMPM_806R_G	GTGACTGGAGTTCAGACGTGTGCTCTTCCGATCTNNNNGGACTACHVGGGTWTCTAAT
JMPM_806R_A	GTGACTGGAGTTCAGACGTGTGCTCTTCCGATCTNNNNAGGACTACHVGGGTWTCTAAT
JMPM_806R_T	GTGACTGGAGTTCAGACGTGTGCTCTTCCGATCTNNNNTAGGACTACHVGGGTWTCTAAT
Adaptor_prim1	AATGATACGGCGACCACCGAGATCTACACGCCTCCCTCGCGCCATCAGAGATGTG
PCR Primer, Index X	CAAGCAGAAGACGGCATACGAGATXXXXXXGTGACTGGAGTTCAGACGTGTGCTC
NextF_Read1_seq	GCCTCCCTCGCGCCATCAGAGATGTGTATAAGAGACAG

## Data Availability

The data presented in this study are available at Qiita at https://qiita.ucsd.edu/, study number 15752. Metabolomics data files are accessible at MetaboLights (accession No. MTBLS10511).

## Supplementary Materials

The following supporting information can be downloaded at https://www.mdpi.com/article/10.3390/nu17091597/s1, Supplementary Tables [Table S1: Denoised Dada2 sequence statistics for ALS; Table S2: Denoised Dada2 sequence statistics for AD; Denoised Dada2 sequence statistics for PD; Table S3: Denoised Dada2 sequence statistics for PD; Table S4: Comprehensive list of metabolites with FC > 1.5 and *p* < 0.1 and/or VIP > 1; Table S5: Biomarkers (metabolites) unique to AD, ALS, and PD]; Supplementary Figures [Figure S1: Metabolites correlated with DHPS in NDDs; Figure S2: Pathways altered in the AD stool metabolome using metabolites with VIP scores > 1; Figure S3: Pathways altered in the ALS stool metabolome using metabolites with VIP scores > 1; Figure S4: Pathways altered in the PD stool metabolome using metabolites with VIP scores > 1; Figure S5: Alpha diversity of stool microbiome in each NDD; Figure S6: Taxa correlated with *Desulfovibrio* in each NDD; Figure S7: Taxa correlated with *Eubacterium* in each NDD; Figure S8: Amyotrophic lateral sclerosis (ALS) sequence quality plots prior to trimming; Figure S9: Alzheimer’s disease (AD) sequence quality plots prior to trimming; Figure S10: Parkinson’s disease (PD) sequence quality plots prior to trimming]; Supplementary Data [Data S1: fold changes *p* value, Data S2: VIP scores, Data S3: DHPS metabolite correlations, Data S4: EdgeR, Data S5: OTU DHPS correlations].
