# Supplementary material for: Specific Bacterial Taxa and Their Metabolite, DHPS, May Be Linked to Gut Dyshomeostasis in Patients with Alzheimer’s Disease, Parkinson’s Disease, and Amyotrophic Lateral Sclerosis
Source: Nutrients. 2025 May 6;17(9):1597. doi: 10.3390/nu17091597 (PMC12073124; doi:10.3390/nu17091597)
Supplement: Supplementary file 1 [file nutrients-17-01597-s001.zip › Supplementary Figures.pdf]

Supplementary Figures

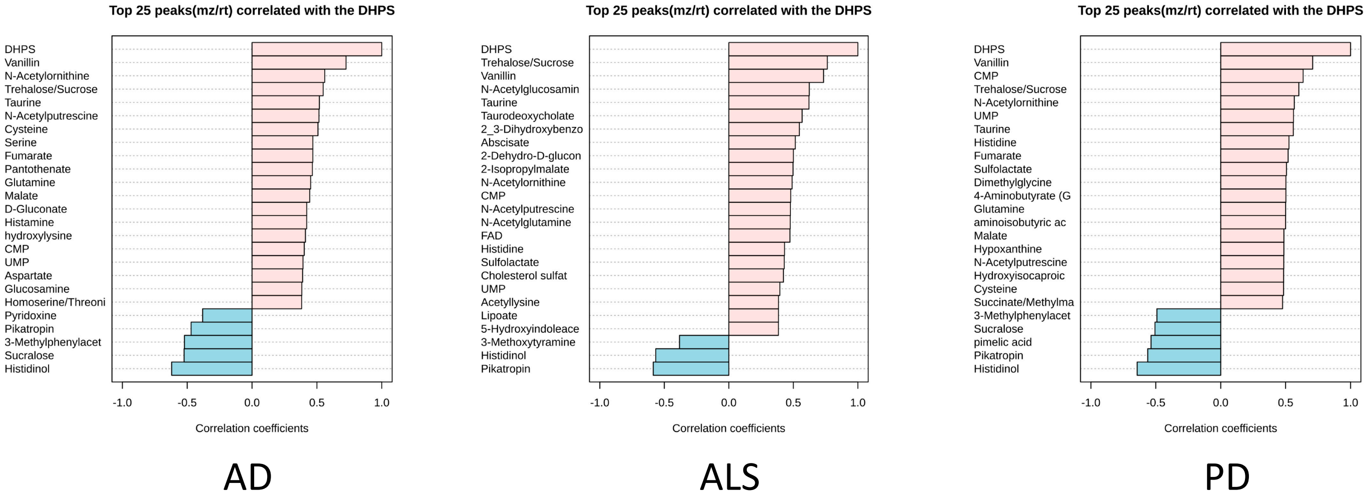

Figure S1. Metabolites correlated with DHPS in NDDs.

AD=Alzheimer's Disease, ALS=amyotrophic lateral sclerosis, PD=Parkinson's Disease
DHPS = 2,3-dihydroxypropane-1-sulfonate, a microbial-derived metabolite
NDDs = neurodegenerative diseases

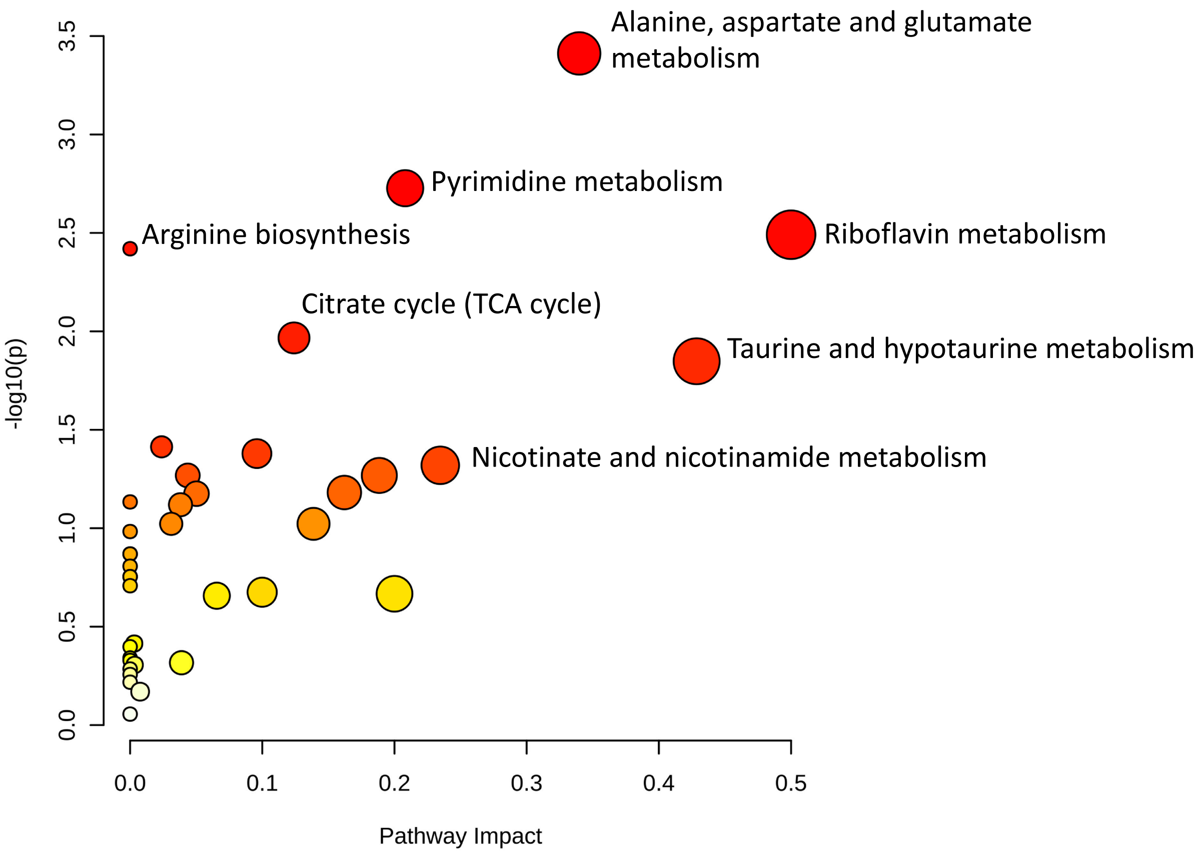

Figure S2. Pathways altered in the AD stool metabolome using metabolites with VIP scores >1.

AD = Alzheimer's Disease, VIP=variable importance projection score

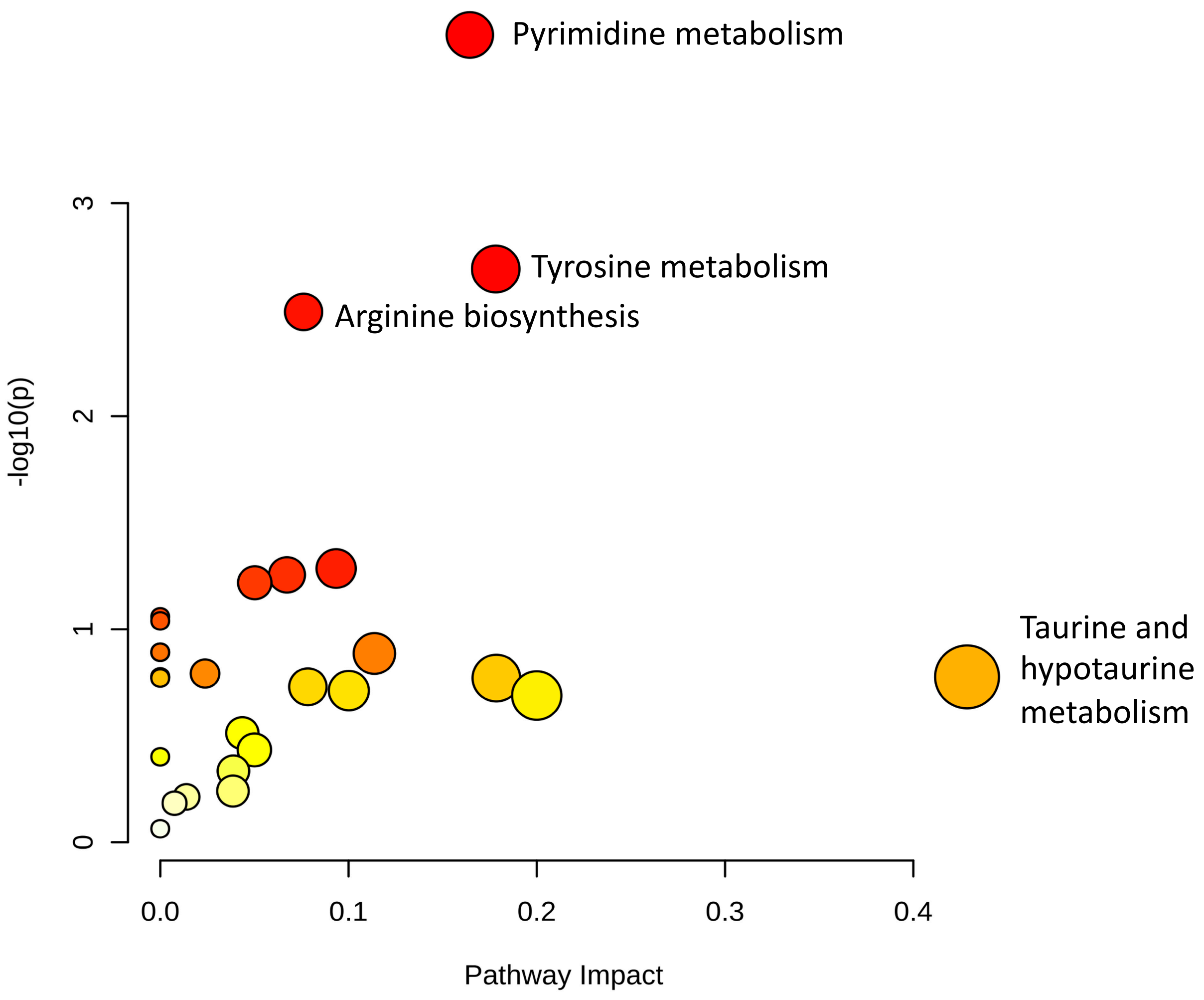

Figure S3. Pathways altered in the ALS stool metabolome using metabolites with VIP scores >1.

ALS = amyotrophic lateral sclerosis; VIP = variable importance projection score

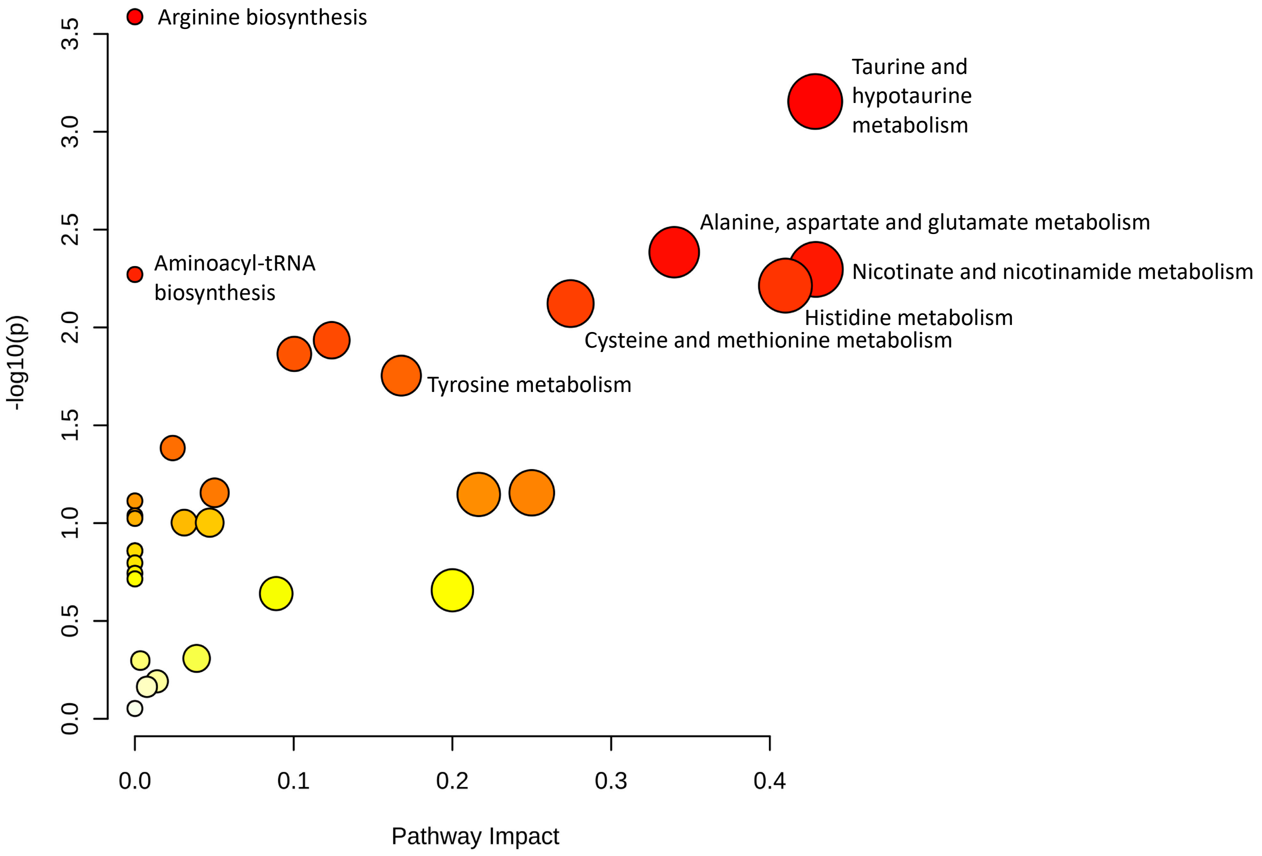

Figure S4. Pathways altered in the PD stool metabolome using metabolites with VIP scores >1.

PD = Parkinson's Disease; VIP = variable importance projection score

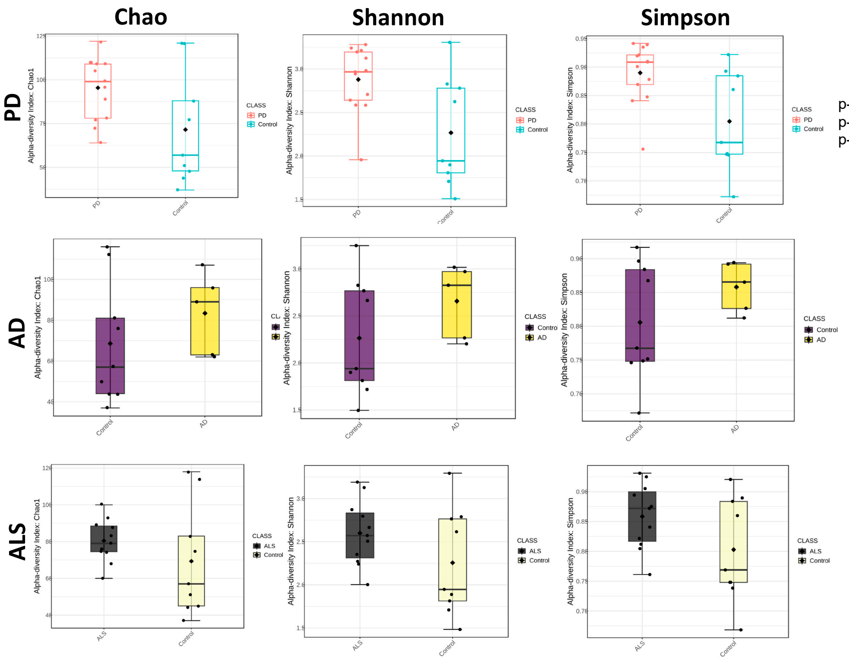

p-value: 0.065993; [Mann-Whitney] statistic: 86.5
p-value: 0.030393; [Mann-Whitney] statistic: 91
p-value: 0.020767; [Mann-Whitney] statistic: 93

p-value: 0.28559; [Mann-Whitney] statistic: 14
p-value: 0.14685; [Mann-Whitney] statistic: 11
p-value: 0.43756; [Mann-Whitney] statistic: 16

p-value: 0.19618; [Mann-Whitney] statistic: 67
p-value: 0.17541; [Mann-Whitney] statistic: 68
p-value: 0.1308; [Mann-Whitney] statistic: 70

Figure S5. Alpha diversity of stool microbiome in each NDD.

NDD = neurodegenerative disease

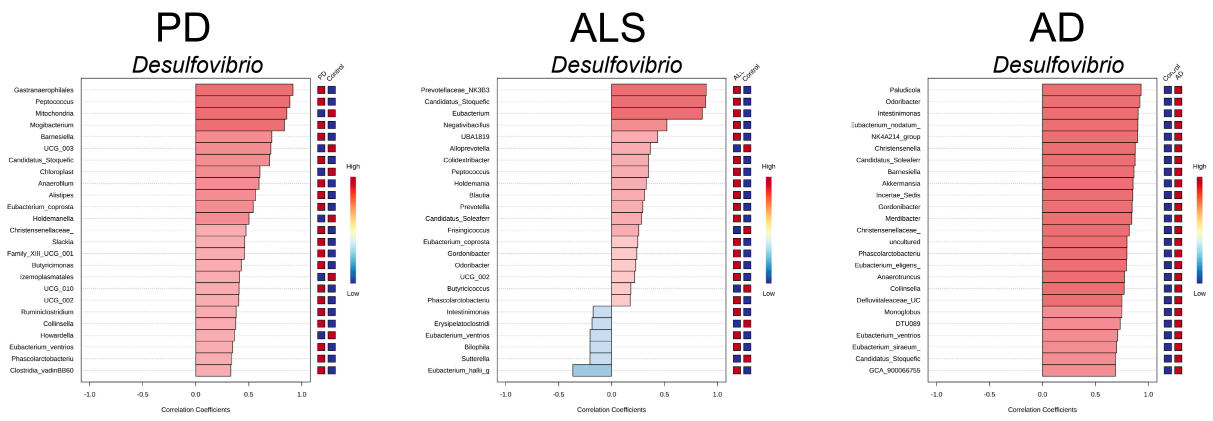

Figure S6. Taxa correlated with *Desulfovibrio* in each NDD.

NDD = neurodegenerative disease

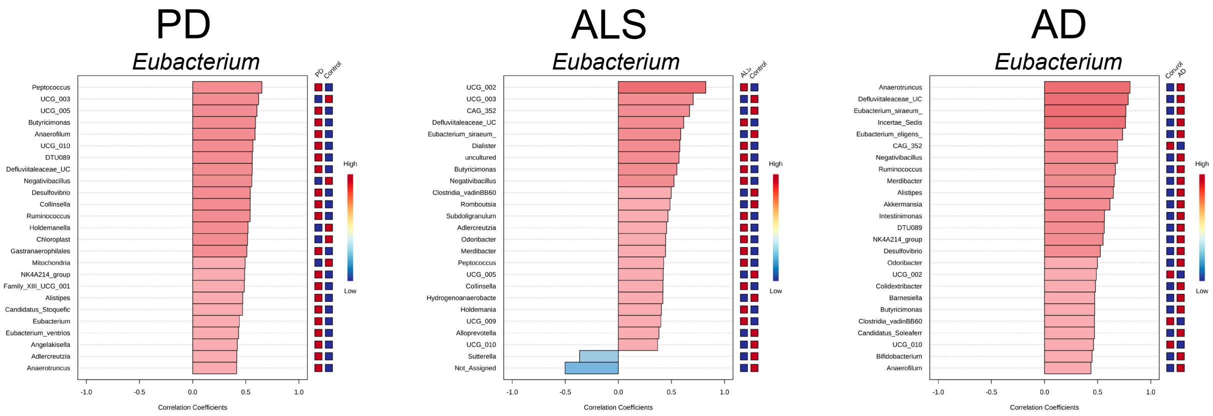

Figure S7. Taxa correlated with *Eubacterium* in each NDD.

NDD = neurodegenerative disease

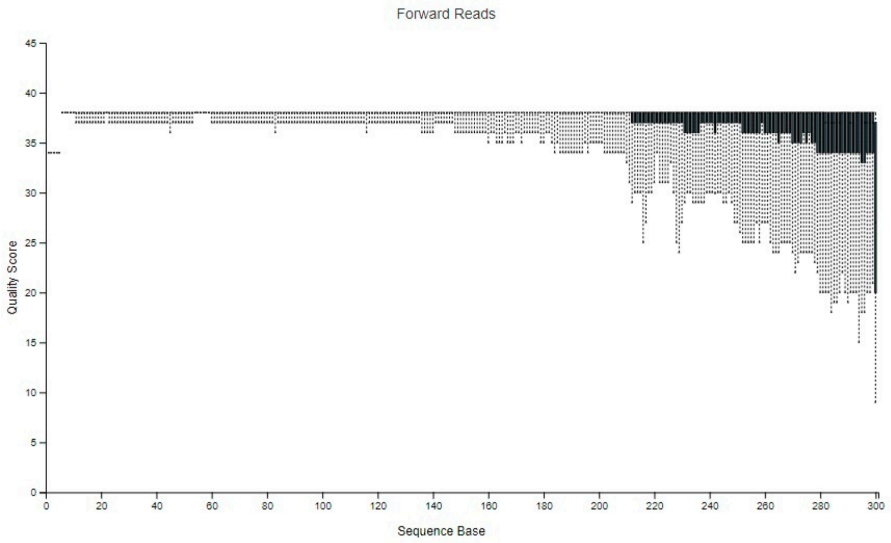

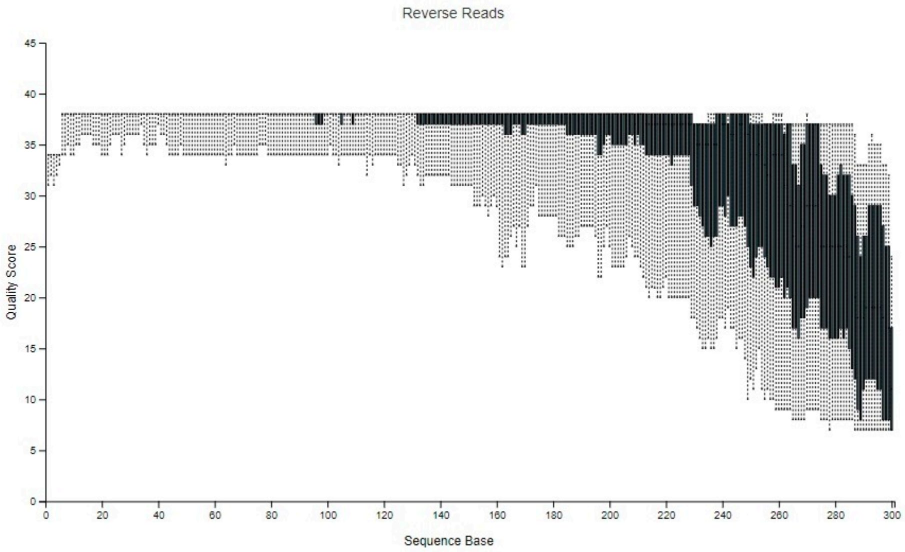

Figure S8: Amyotrophic lateral sclerosis (ALS) sequence quality plots prior to trimming. For ALS, p-trim-left was set to 20 and p-trim-len was set to 220.

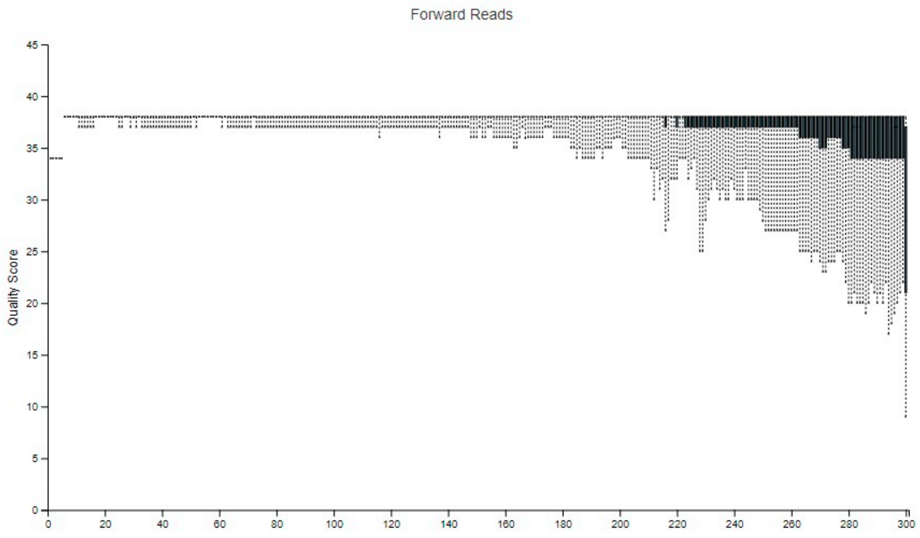

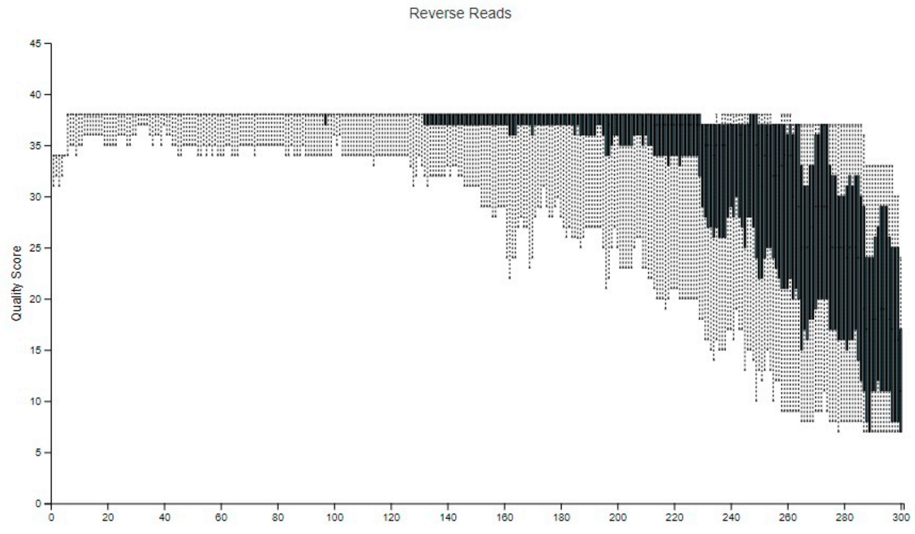

Figure S9: Alzheimer's Disease (AD) sequence quality plots prior to trimming. For AD, p-trim-left was set to 20 and p-trim-len was set to 220.

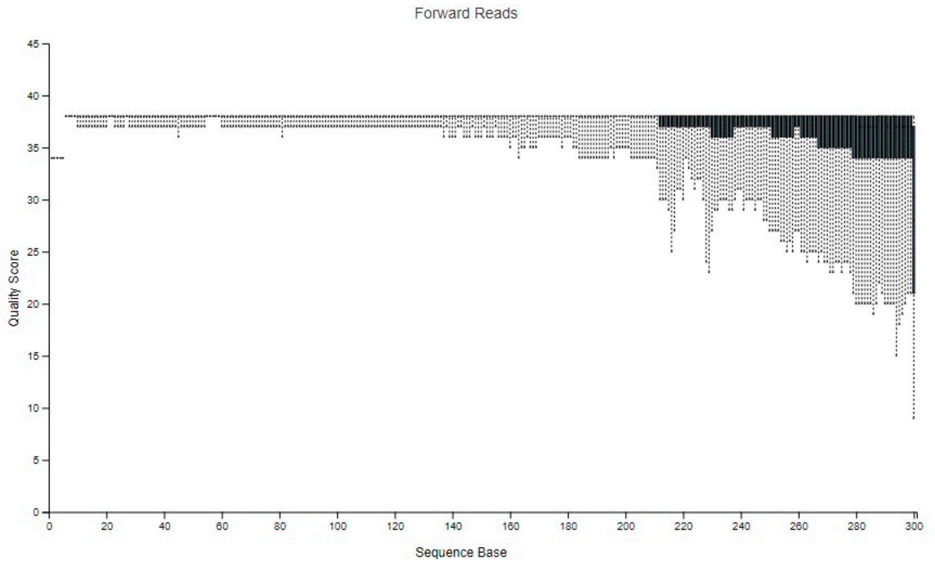

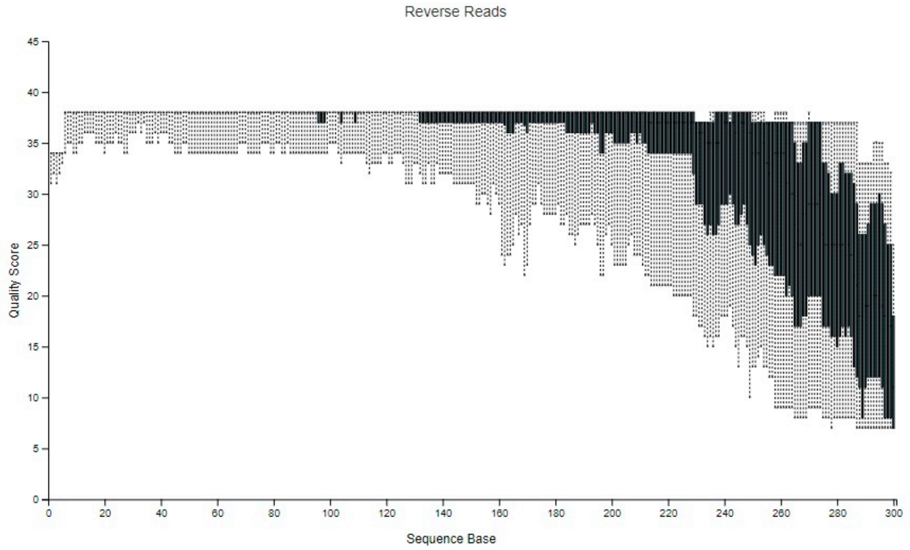

Figure S10: Parkinson's Disease (PD) sequence quality plots prior to trimming. For PD, p-trim-left was set to 20 and p-trim-len was set to 220.
