# Supplementary material for: Specific Bacterial Taxa and Their Metabolite, DHPS, May Be Linked to Gut Dyshomeostasis in Patients with Alzheimer’s Disease, Parkinson’s Disease, and Amyotrophic Lateral Sclerosis
Source: Nutrients. 2025 May 6;17(9):1597. doi: 10.3390/nu17091597 (PMC12073124; doi:10.3390/nu17091597)
Supplement: Supplementary file 1 [file nutrients-17-01597-s001.zip › Supplementary Tables.pdf]

Supplementary Tables

Table S1: Denoised Dada2 sequence statistics for ALS.

sample-id	input	filtered	percentage of input passed filter	denoised	non-chimeric	percentage of input non-chimeric
UT001	3333341	3223084	96.69	3215930	2675917	80.28
UT002	264625	248521	93.91	247619	230705	87.18
UT004	146448	130887	89.37	130530	123611	84.41
UT005	289498	272664	94.19	271792	246491	85.14
UT007	332208	310075	93.34	309144	291650	87.79
UT009	304345	285062	93.66	284213	267530	87.9
UT021	283630	266267	93.88	265069	248373	87.57
UT022	315324	296465	94.02	295263	280083	88.82
UT024	281557	264174	93.83	263344	250738	89.05
UT025	368934	345718	93.71	344637	316612	85.82
UT026	371513	349092	93.96	347847	328903	88.53
UT027	239266	145559	60.84	144093	139183	58.17
UT029	271310	254847	93.93	253717	231167	85.2
UT030	277108	261399	94.33	260379	231942	83.7
UT031	339879	320361	94.26	319653	290788	85.56
UT033	400676	375729	93.77	374342	348760	87.04
UT035	304667	287292	94.3	286415	267373	87.76
UT038	282330	263854	93.46	263209	247574	87.69
UT043	337674	304102	90.06	302675	283800	84.05
UT044	240100	218642	91.06	216363	209978	87.45
UT045	379638	354653	93.42	352564	340359	89.65

ALS = amyotrophic lateral sclerosis

Table S2: Denoised Dada2 sequence statistics for AD.

sample-id	input	filtered	percentage of input passed filter	denoised	non-chimeric	percentage of input non-chimeric
UT001	3333341	3223084	96.69	3215930	2527333	75.82
UT004	146448	130887	89.37	130530	124618	85.09
UT005	289498	272664	94.19	271792	244145	84.33
UT024	281557	264174	93.83	263344	244656	86.89
UT031	339879	320361	94.26	319653	290788	85.56
UT033	400676	375729	93.77	374342	339801	84.81
UT036	319972	300338	93.86	299350	275837	86.21
UT038	282330	263854	93.46	263209	244238	86.51
UT039	320994	301967	94.07	301137	285524	88.95
UT040	331360	311782	94.09	310839	280192	84.56
UT041	277539	261496	94.22	260403	236380	85.17
UT042	292256	275274	94.19	274322	257175	88
UT043	337674	304102	90.06	302675	278357	82.43
UT044	240100	218642	91.06	216363	204172	85.04
UT045	379638	354653	93.42	352564	341915	90.06

AD = Alzheimer’s Disease

Table S3: Denoised Dada2 sequence statistics for PD.

sample-id	input	filtered	percentage of input passed filter	denoised	non-chimeric	percentage of input non-chimeric
UT001	3333341	3223084	96.69	3215930	2689852	80.7
UT003A	327352	308036	94.1	306966	295930	90.4
UT004	146448	130887	89.37	130530	126167	86.15
UT005	289498	272664	94.19	271792	246133	85.02
UT011	402573	378535	94.03	377640	360867	89.64
UT012	331555	311317	93.9	310091	286317	86.36
UT013	386888	360794	93.26	359608	338338	87.45
UT014	349287	327731	93.83	326512	306906	87.87
UT015	371556	350038	94.21	348850	339371	91.34
UT016	348217	328968	94.47	328013	303594	87.19
UT018	342045	321908	94.11	320906	298046	87.14
UT019	305939	287747	94.05	286866	279062	91.21
UT023	378100	355478	94.02	354324	340951	90.17
UT024	281557	264174	93.83	263344	248938	88.41
UT031	339879	320361	94.26	319653	291581	85.79
UT033	400676	375729	93.77	374342	349541	87.24
UT038	282330	263854	93.46	263209	247674	87.73
UT043	337674	304102	90.06	302675	286359	84.8
UT044	240100	218642	91.06	216363	203626	84.81
UT045	379638	354653	93.42	352564	340822	89.78
UT060	518396	468026	90.28	466205	455960	87.96
UT061	397674	372754	93.73	371220	361173	90.82
UT063	348004	323784	93.04	322503	314395	90.34

PD = Parkinson’s Disease

Table S4. Comprehensive List of Metabolites with FC>1.5 and p<0.1 and/or VIP>1

AD	ALS	PD
DHPS	N-Acetylglucosamine 1/6-phosphate	Trehalose/Sucrose
Fumarate	Cholesterol sulfate	2-Isopropylmalate
Malate	2-Isopropylmalate	Phenyllactic acid
4-Pyridoxate	Xanthurenic acid	5-Hydroxyindoleacetic acid (5-HIAA)
Lipoate	Kynurenic acid	Sulfolactate
UMP	Vanillin	Histidine
Biotin	DHPS	2-Hydroxy-2-methylsuccinate
IMP	Glycodeoxycholate	Lipoate
Deoxyribose phosphate	N-Acetylglutamine	2-hydroxyglutaric acid
Cysteine	Sulfolactate	2-Dehydro-D-gluconate
D-Gluconate	2_3-Dihydroxybenzoate	UMP
Aspartate	Lipoate	CMP
Taurine	3_4-Dihydroxyphenylacetate (DOPAC)	DHPS
FAD	5-Hydroxyindoleacetic acid (5-HIAA)	D-Gluconate
Histamine	D-Glucarate	N-Acetylglucosamine 1/6-phosphate
hydroxylysine	N-Acetylornithine	Biotin
Glucosamine phosphate	Pyridoxine	Cytidine
S-Ribosyl-L-homocysteine	Aconitate	3-Methylthiopropionate
CMP	2-Dehydro-D-gluconate	Fumarate
N-Acetylglucosamine 1/6-phosphate	Jasmonate	Hypoxanthine
homocarnosine	Taurodeoxycholate	Aspartate
Vanillin	FAD	Gluconolactone
Pikatropin	Sedoheptulose 1/7-phosphate	xylose
3-Methylthiopropionate	Creatine	Cholesterol sulfate
Hypoxanthine	sn-Glycerol 3-phosphate	N-Acetylornithine
Aconitate	UMP	Glutamine
Histidinol	Dopamine	N-Acetylglucosamine
Serine	2-Hydroxy-2-methylsuccinate	Vanillin
D-Glyceraldehdye 3-phosphate	Creatinine	Dopamine
Glycerone phosphate	Homovanillic acid (HVA)	glutaric acid
dTMP	2-hydroxyglutaric acid	methyl succinic acid
Homovanillic acid (HVA)	CMP	Homovanillic acid (HVA)
Cholesterol sulfate	Trehalose/Sucrose	N-Acetyl-beta-alanine
2-Dehydro-D-gluconate	methyl succinic acid	Malate
3-Methylphenylacetic acid	glutaric acid	Aconitate
Glutamine	Glutamine	Histidinol
Guanine	N-Acetylputrescine	Taurine
N-Carbamoyl-L-aspartate	Asparagine	Cystathionine

Trehalose/Sucrose	Orotate	3_4-Dihydroxyphenylacetate (DOPAC)
sn-Glycerol 3-phosphate	Cytidine	Lysine
D-Glucarate	N-Acetylglucosamine	NAD+
Sulfolactate	Histidinol	Xylitol
Carnitine	Taurine	Serine
Phenyllactic acid	CDP-choline	Sucralose
FMN	Guanosine	IMP
Sucralose	Cystathionine	Histamine
NAD+	Biotin	Taurodeoxycholate
	Pikatropin	GMP
	Arginine	Hydroxyisocaproic acid
	Uridine	3-Hydroxyisovalerate
	GMP	pimelic acid
	3-Methoxytyramine (3-MT)	Cysteate
	Hydroxyisocaproic acid	Pikatropin
	3-Methylthiopropionate	Cysteine
	Ribose phosphate	Nicotinamide
	Abscisate	Asparagine
		FAD

AD=Alzheimer's Disease, ALS=amyotrophic lateral sclerosis, and PD=Parkinson's Disease; FC = fold change; VIP = variable importance in projectionscore based on partial least-squares-discriminant analysis (PLS-DA)

Table S5. Biomarkers (Metabolites) Unique to AD, ALS, and PD

Unique to ALS	Unique to AD	Unique to PD	Conserved in ALS, AD, PD	ALS and PD	ALS and AD	AD and PD
Kynurenic acid	hydroxylysine	Histidine	N-Acetylglucosamine 1/6-phosphate	2-Isopropylmalate	D-Glucarate	IMP
Xanthurenic acid	4-Pyridoxate	Gluconolactone	Cholesterol sulfate	Taurodeoxycholate	sn-Glycerol 3-phosphate	Histamine
2_3-Dihydroxybenzoate	Deoxyribose phosphate	xylose	DHPS	5-Hydroxyindoleacetic acid (5-HIAA)		Cysteine
Glycodeoxycholate	Glucosamine phosphate	3-Hydroxyisovalerate	CMP	Cystathionine		Malate
Sedoheptulose 1/7-phosphate	S-Ribosyl-L-homocysteine	pimelic acid	Vanillin	N-Acetylornithine		Fumarate
Creatine	homocarnosine	N-Acetylglucosamine	Aconitate	Asparagine		D-Gluconate
Creatinine	Serine	Cysteate	UMP	Cytidine		Hypoxanthine
CDP-choline	D-Glyceraldehdye 3-phosphate	Xylitol	Sulfolactate	Dopamine		Aspartate
N-Acetylglutamine	Glycerone phosphate	Nicotinamide	Histidinol	2-Hydroxy-2-methylsuccinate		Phenyllactic acid
Guanosine	dTMP	Lysine	Taurine	GMP		Sucralose
DOPAC	3-Methylphenylacetic acid		Homovanillic acid (HVA)	2-hydroxyglutaric acid		
Orotate	Guanine		FAD	Hydroxyisocaproic acid		
N-Acetylputrescine	N-Carbamoyl-L-aspartate		2-Dehydro-D-gluconate	methyl succinic acid		
Jasmonate	Carnitine		Biotin	glutaric acid		
Arginine	FMN		Lipoate			
Uridine			Pikatropin			
3-Methoxytyramine (3-MT)			Glutamine			
Pyridoxine			3-Methylthiopropionate			
Ribose phosphate			Trehalose/Sucrose			
Abscisate						

[AD=Alzheimer's Disease, ALS=amyotrophic lateral sclerosis, and PD=Parkinson's Disease]
